# Cell Context Dependent p53 Genome-Wide Binding Patterns and Enrichment at Repeats

**DOI:** 10.1371/journal.pone.0113492

**Published:** 2014-11-21

**Authors:** Krassimira Botcheva, Sean R. McCorkle

**Affiliations:** Biosciences Department, Brookhaven National Laboratory, Upton, NY, 11973, United States of America; Georgia Regents University, United States of America

## Abstract

The p53 ability to elicit stress specific and cell type specific responses is well recognized, but how that specificity is established remains to be defined. Whether upon activation p53 binds to its genomic targets in a cell type and stress type dependent manner is still an open question. Here we show that the p53 binding to the human genome is selective and cell context-dependent. We mapped the genomic binding sites for the endogenous wild type p53 protein in the human cancer cell line HCT116 and compared them to those we previously determined in the normal cell line IMR90. We report distinct p53 genome-wide binding landscapes in two different cell lines, analyzed under the same treatment and experimental conditions, using the same ChIP-seq approach. This is evidence for cell context dependent p53 genomic binding. The observed differences affect the p53 binding sites distribution with respect to major genomic and epigenomic elements (promoter regions, CpG islands and repeats). We correlated the high-confidence p53 ChIP-seq peaks positions with the annotated human repeats (UCSC Human Genome Browser) and observed both common and cell line specific trends. In HCT116, the p53 binding was specifically enriched at LINE repeats, compared to IMR90 cells. The p53 genome-wide binding patterns in HCT116 and IMR90 likely reflect the different epigenetic landscapes in these two cell lines, resulting from cancer-associated changes (accumulated in HCT116) superimposed on tissue specific differences (HCT116 has epithelial, while IMR90 has mesenchymal origin). Our data support the model for p53 binding to the human genome in a highly selective manner, mobilizing distinct sets of genes, contributing to distinct pathways.

## Introduction

Ever since the ability of the p53 tumor suppressor protein to bind DNA in a sequence-specific manner was established [1],[2], the specificity of p53 genomic binding has been intensively studied to gain insight into the network of p53 dependent target genes and their role in ensuring genomic protection and tumor suppression [3]. Currently, there are about 200 individually validated p53 binding sites [4]–[6] and thousands identified by genome-wide studies [7]–[15]. Previously, we reported significant differences between the genome-wide distributions of the p53 binding sites we mapped in normal human fibroblasts [16] and those identified by others in human cancer cell lines [8],[11],[12]. Direct comparison and interpretation of differences between non-coordinated genome-wide studies is challenging when experimental variations are considered (e.g. cell types, treatment conditions, and sequencing approaches). The four datasets we analyzed previously [16] differed by biological origin, throughput and resolution; two were generated using the same approach (ChIP-seq) and different cell lines/treatment conditions (IMR90/6 hrs 5 FU; U2OS/24 hrs Actinomycin), and two were performed under the same conditions (6 hrs, 5 FU) using different cell lines/sequencing approaches (IMR90/ChIP-seq; HCT116/ChIP-PET). To eliminate experimental variations as a contributing factor for the p53 binding differences, we set to examine the p53 genome-wide binding in datasets that differ only by the cell lines in which they were generated. We mapped *de novo* the p53 binding sites in the cancer cell line HCT116, applying the same treatment (6 hrs, 5-FU), approach (ChIP-seq) and analysis pipeline, used for our IMR90 study. We report here, that the p53 binding sites reside in distinct genomic landscapes in the HCT116 and IMR90 cell lines, under the same experimental conditions. That result confirms p53 binding to the genome in a cell context-dependent manner.

## Results and Discussion

### ChIP-seq map of p53 genomic binding sites in HCT116 cells

We applied high-throughput sequencing of chromatin immunoprecipitated DNA (ChIP-seq) and mapped the endogenous wild type p53 genomic binding sites in HCT116 cells, treated with 5-FU for 6 hrs. These conditions allowed a direct comparison with our previous IMR90 p53 ChIP-seq study [16] (same approach, different cell lines), and with the HCT116 p53 ChIP-PET study [8] (same cell lines and treatments, different approaches, ChIP-seq vs ChIP-PET). Treatment with 5-FU for 6 hrs led to significant p53 induction in the HCT116 cells (Figure S1, panel A). While in the untreated IMR90 cells there was little p53 protein detected, the untreated HCT116 cells contained a considerable amount, probably due to constitutive p53 activation [17]. Before preparing the sequencing libraries we verified p53 binding at some known target sites, including *CDKN1A* and *MDM2* (Figure S1, panel B). A p53-specific ChIP library obtained with the DO1 antibody and Input library (chromatin sample taken before ChIP) were sequenced on Illumina GA 2x, generating 13.7 million ChIP-seq and 11.6 million Inp-seq reads (Table S1). More than 88% of the obtained reads were mapped back to the human genome (hg18), and the distinct sequences mapped uniquely were used for further analysis and peak definition. We used a peak finding method based on the approach of Rozowsky et al. [18], as described previously [16], and identified total of 3,750 p53 ChIP-seq peaks and 2,168 Inp-seq peaks (Figure S2). To define a set of high-confidence ChIP-seq peaks enriched above the Input-seq control (chromatin before the immunoprecipitation), and most likely representing p53 binding sites, we applied two independent confidence tests, as described [16]. A minimum score of 99% was required in both, to call a peak high-confident. The 550 high-confident ChIP-seq peaks are listed in Table S2.

**Figure 1:**
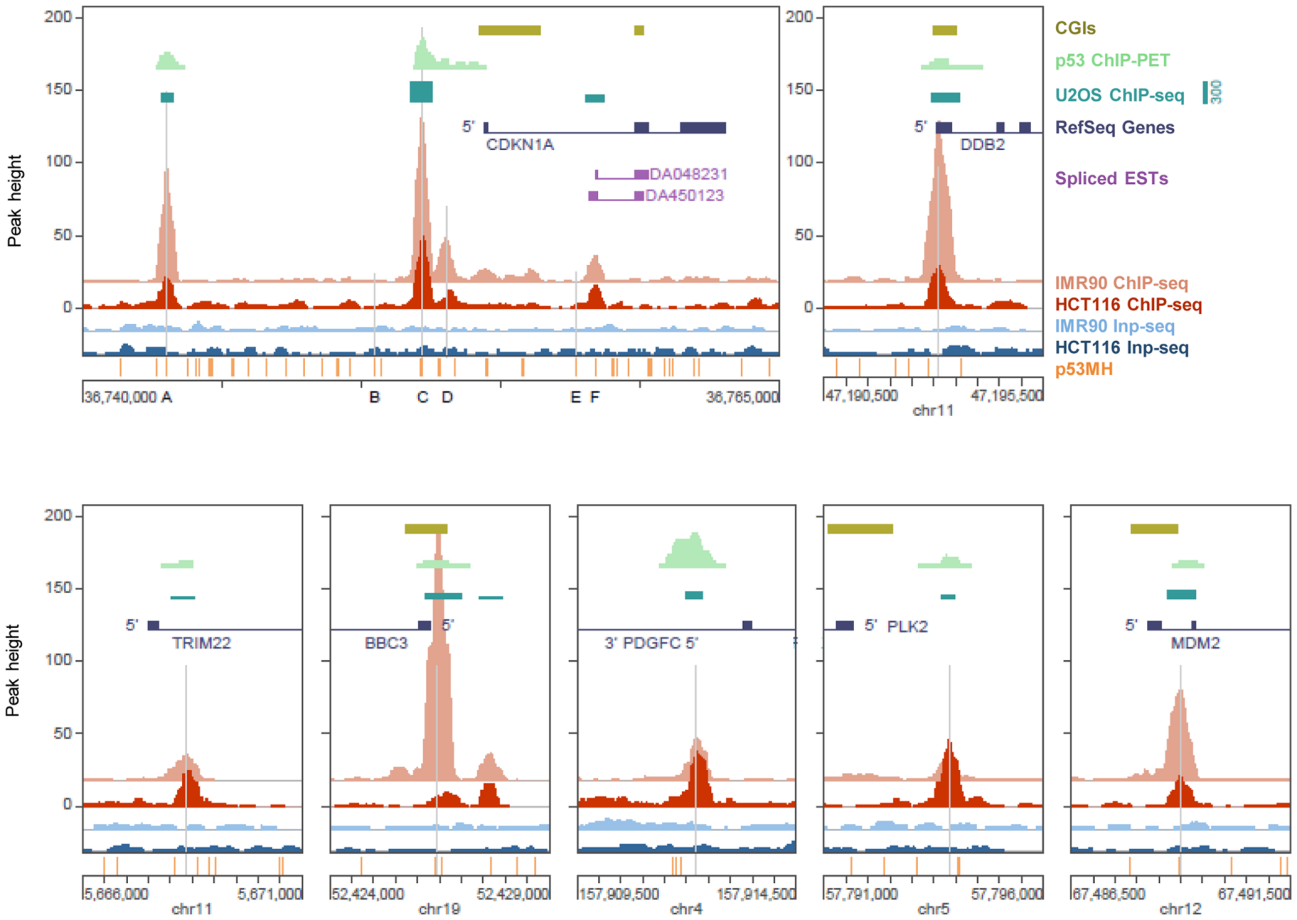
p53 ChIP-seq maps in HCT116 cells at the *CDKN1A* locus (25 kb window), and at the target genes DDB2, TRIM22, BBC3, PDGFC, PLK2 and MDM2 (5 kb window). Plotted are coverage maps of ChIP-seq (dark red) and Input-seq (dark blue). Reference p53 binding sites are marked with vertical grey lines, sites predicted by p53MH algorithm [21] are shown at the bottom of the panels. Previously reported data plotted for comparison: p53 ChIP-seq (light red) and Inp-seq (light blue), IMR90 [16], p53 ChIP-PET, HCT116 [8] and p53 ChIP-seq, U2OS cells [12]. Other genomic features shown: CpG islands (CGI), RefSeq genes, expressed sequencing tags (ESTs), as annotated, UCSC Human Genome Browser, hg18. Same y-axis scale applied to HCT116 and IMR90 ChIP-seq and Inp-seq; baseline shown for the HCT116 ChIP-seq set (the others shifted for easier viewing); same datasets color scheme followed for the rest of the paper.

Examples of high-confidence p53 ChIP-seq peaks identified in the HCT116 cells are shown in Figure 1. Plotted for comparison are HCT116 ChIP-PET clusters [8], IMR90 ChIP-seq peaks [16], and U2OS ChIP-seq peaks [12]. p53 binding at the *CDKN1A* locus was consistent in all datasets shown; highest binding at site C, followed by binding at site A and site F (Figure 1). However, that was not the case at most of the targets; for example at *PDGFC* and *PLK2*, p53 binding in HCT116 was noticeably stronger than in IMR90 cells, while at *DDB2*, *MDM2* and *BBC3/PUMA* it was the opposite (Figure 1). In most cases of weaker p53 binding in HCT116, the affected sites resided at CpG islands (CGIs). At targets like *BBC3/PUMA*, with two p53 binding sites in a close proximity [19],[20], relative changes in p53 enrichment can be observed (the site with higher p53 binding in IMR90 becoming the site with lower p53 binding in HCT116, Figure 1). Such opposite trends of p53 enrichment in HCT116 and IMR90 cells were noticeable at many targets, particularly at sites residing at, or in close proximity to CGIs.

### Cell context specific p53 genomic binding

**Figure 2:**
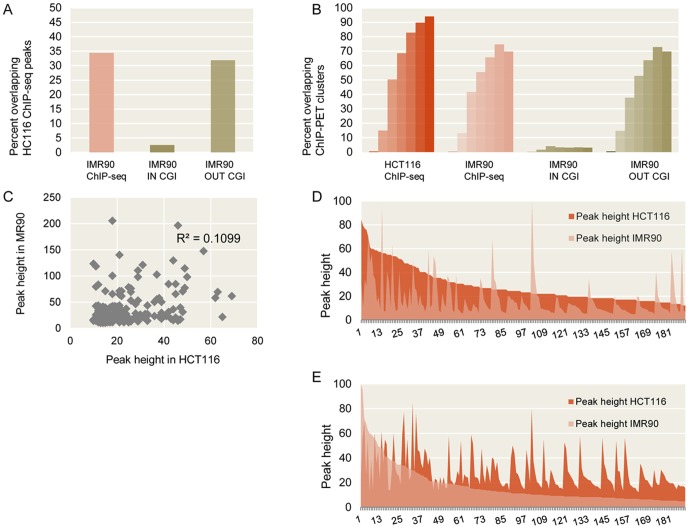
Comparison of high-confidence p53 ChIP-seq peaks identified in HCT116 (this study) and IMR90 [16], with ChIP-PET clusters reported in HCT116 cells [8]. A) The overlap between the p53 ChIP-seq peaks in HCT116 and IMR90 is due mainly to the fraction of IMR90 peaks outside CpG islands. B) The overlap of HCT116 p53 ChIP-PET clusters with HCT116 p53 ChIP-seq peaks is higher than that with the IMR90 p53 ChIP-seq peaks. ChIP-PET clusters reported by rank (PET1+ to PET7+) are plotted with increasing color intensity. C) No correlation between ChIP-seq peak heights in HCT116 (x-axis) and IMR90 (y-axis) at the 189 sites identified in both cell lines. D) and E) No correlation between p53 ChIP-seq peak heights in the two cell lines after ordering by peak height either in HCT116 (D), or in IMR90 (E), see also Materials and Methods.

We reasoned that if the p53 genome-wide binding is selective and cell context dependent, our *de novo* obtained HCT116 p53 ChIP-seq dataset should agree better with the HCT116 p53 ChIP-PET [8] than with the IMR90 ChIP-seq [16] dataset. Therefore, we compared the three studies. About 35% of the high-confidence p53 ChIP-seq peaks identified in HCT116 were also mapped in IMR90 (Table S3). This overlap was due predominantly to the fraction of IMR90 peaks out of CGIs, with very little contribution from IMR90 peaks in CGIs (Figure 2A). Since the ChIP-PET data are statistically different from the ChIP-seq, in order to compare them, the ChIP-PET clusters were reported as originally defined [8], by rank (PET1+ to PET7+), see also Materials and Methods. There was a significantly higher overlap of HCT116 ChIP-PET clusters with HCT116 ChIP-seq peaks, than with IMR90 ChIP-seq peaks (Figure 2B). Although higher rank ChIP-PET clusters showed better overlap with both datasets (statistically expected), beyond that general trend, the strong ChIP-PET clusters were almost completely identified by the HCT116 ChIP-seq peaks, but not by the IMR90 ChIP-seq peaks. More than 30% of the HCT116 ChIP-PET7+ clusters were not identified by high-confidence IMR90 ChIP-seq peaks, compared to only ∼5% not identified by high-confidence HCT116 ChIP-seq peaks. Of note, the reason for the latter was not absence of detected p53 binding, on the contrary, strong HCT116 ChIP-seq peaks were observed at these locations, but presence of highly symmetrical overlapping Inp-seq peaks disqualified them from the high-confidence set. Interestingly, compared to ChIP-PET6+, the higher rank ChIP-PET7+ clusters show lower overlap by IMR90 ChIP-seq peaks, contrary to statistical expectations (better overlap for higher ranked clusters), with the possible explanation that the binding sites with highest p53 occupancy represent distinct sets in the two cell lines.

Next, we asked if p53 was similarly enriched at the sites common for the two cell lines (189 ChIP-seq peaks, Table S3). There was no correlation between the peak heights in HCT116 and IMR90 at the common sites (R^2^ = 0.1099, Figure 2C), unlike the high correlation observed between the common Inp-seq peaks in the two cell lines (R^2^ = 0.92, data not shown). No correlation was seen upon ordering the 189 p53 ChIP-seq peaks, either by peak height in HCT116 (Figure 2D), or by peak height in IMR90 (Figure 2E), see also Materials and Methods. Despite the different experimental approaches, the HCT116 ChIP-seq and HCT116 ChIP-PET p53 binding studies agree better than the HCT116 ChIP-seq and IMR90 ChIP-seq studies, which despite the same experimental conditions reveal significant differences, pointing at cell context dependent p53 genomic binding.

### Genomic landscape of the p53 binding sites

**Figure 3:**
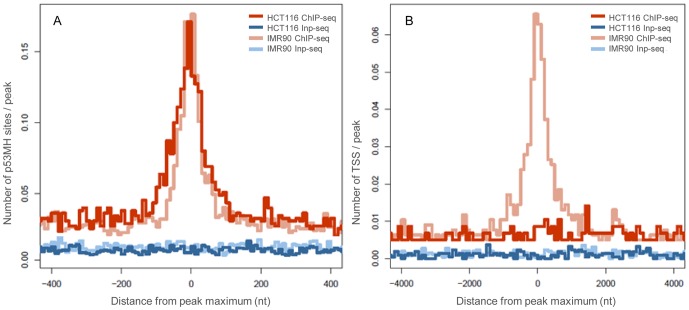
Distribution of predicted p53 binding sites (p53MH) and transcription start sites (TSS) with respect to high-confidence p53 ChIP-seq peaks in HCT116. Shown for comparison are IMR90 data [16]. A) Enrichment of predicted p53 sites defined by p53MH algorithm [21] at p53 ChIP-seq peaks in both cell lines. B) No TSS enrichment at HCT116 ChIP-seq, unlike at IMR90 ChIP-seq peaks. Similar distribution at Inp-seq sets.

The p53 binding sites in HCT116 and IMR90 cell lines resided in different genomic landscapes. Both high-confidence ChIP-seq datasets showed similar enrichment for predicted p53 binding sites at the peak maxima, as defined by the p53MH algorithm [21], indicating similar likelihood for containing p53-bound sequences. As expected, the control Inp-seq datasets did not show such enrichment (Figure 3A). In the HCT116 cells there was no p53 ChIP-seq peaks enrichment at transcription start sites (TSS, Figure 3B), unlike in the IMR90 ChIP-seq [16]. Although at present the functional significance of the p53 binding differences with respect to TSS are not understood, it is unlikely they result from random events. Recent data from human embryonic stem cells undergoing differentiation or DNA damage revealed that 27% of the p53 binding sites associated with differentiation, while 13% of the sites associated with DNA damage, resided within 1 kb of TSSs [22]. Different proximities to TSSs might reflect differences in the type of transcriptional control evoked by p53 at functionally distinct groups of targets.

**Figure 4:**
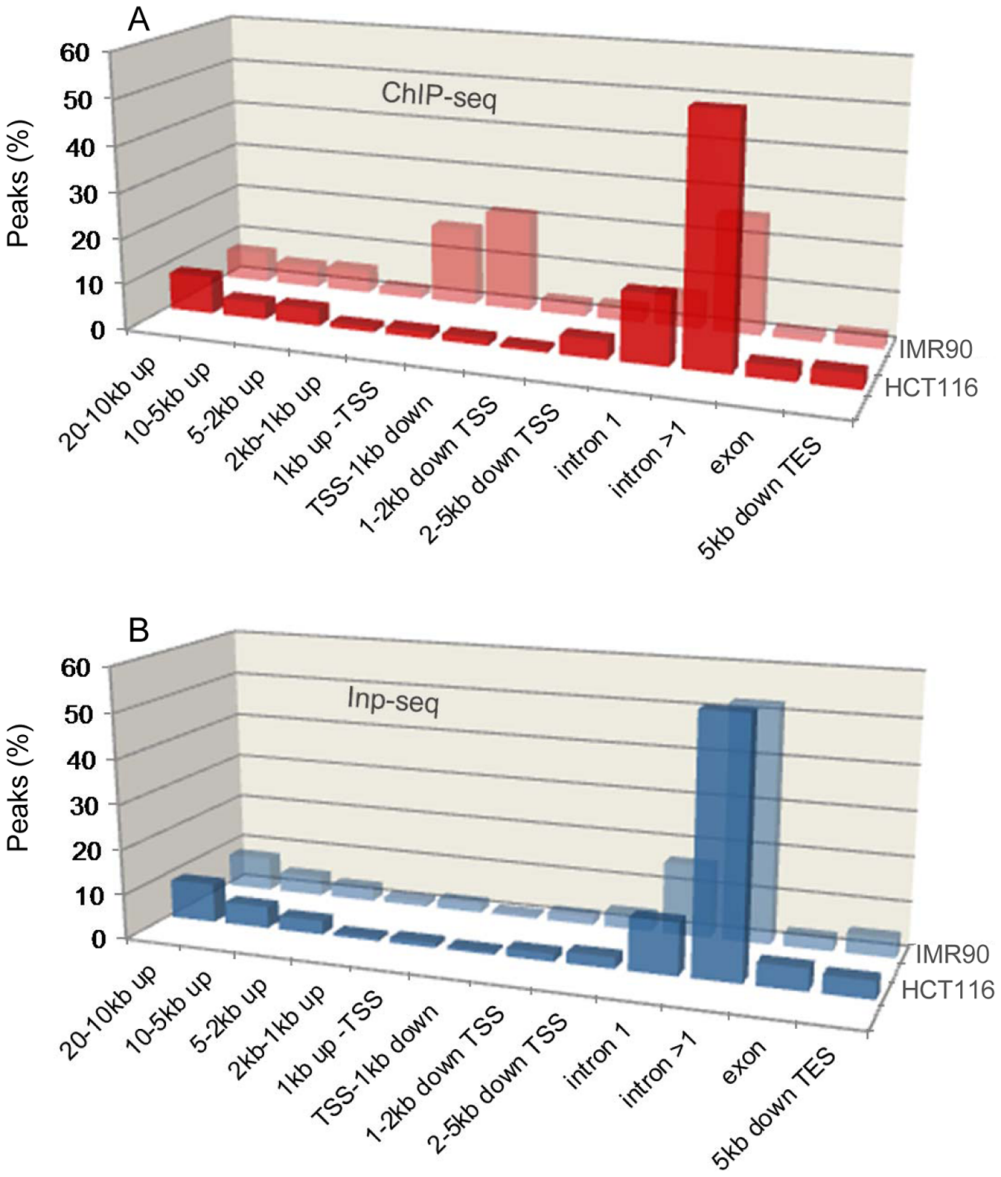
High-confidence p53 ChIP-seq peaks in HCT116 and IMR90 cells [16] show different distributions with respect to genes, unlike Inp-seq sets from the two cell lines. Plotted are fractions of peaks in the non-overlapping genic regions: 20–10 kb to TSS; 10–5 kb to TSS; 5–2 kb to TSS; 2–1 kb to TSS; 1 kb to TSS; TSS to 1 kb down; 1–2 kb down TSS; 2–5 kb down TSS; intron 1; intron >1; exon; 5 kb down TES (transcription end site). Peaks are reported in intron1, intron >1 or exon, only if located more than 5 kb downstream of TSS, otherwise their exact distance to TSS is reported. See also Table S4.

Given the absence of TSS enrichment at the HCT116 p53 ChIP-seq peaks, we examined their proximity to genes, determining the fractions of high-confidence peaks in regions spanning from 20 kb upstream of TSS to 5 kb downstream of transcription end site (TES), Figure 4 and Table S4, see also Materials and Methods. Although enriched p53 binding in the first intron has been reported, here the higher fraction of HCT116 p53 ChIP-seq peaks in introns (including the first intron), does not appear p53 specific, since it is present in the Inp-seq control, reflecting the large size of these regions. Plotted for comparison are the IMR90 ChIP-seq peaks [16] showing specific enrichment within 1–2 kb of TSS (not present in the control Inp-seq, Figure 4).

**Figure 5:**
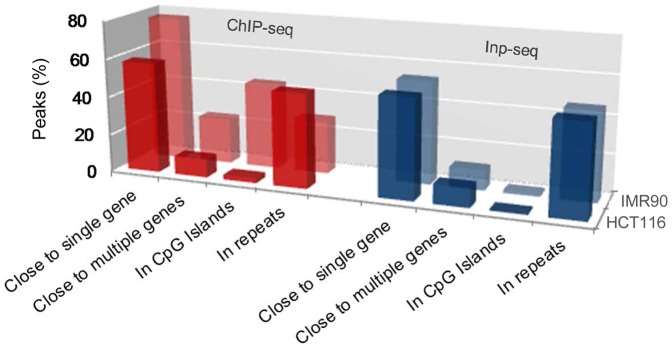
High-confidence p53 ChIP-seq peaks in HCT116 cells are less enriched in proximity to genes, depleted at CpG islands, and more enriched at repeats, compared to IMR90 [16]. Inp-seq peaks from both cell lines show similar distributions.

The p53 binding sites in the HCT116 and IMR90 cell lines reside in genomic regions with different gene density. While a significant fraction of the HCT116 p53 ChIP-seq peaks was found associated with a single gene/transcript (∼50%), it was lower compared to the IMR90 ChIP-seq peaks (∼70%), Figure 5. The difference was more obvious when multi-genic regions were considered. The fraction of p53 ChIP-seq peaks in proximity to multiple genes in HCT116 was less than half of that in IMR90 cells, see Materials and Methods.

**Figure 6:**
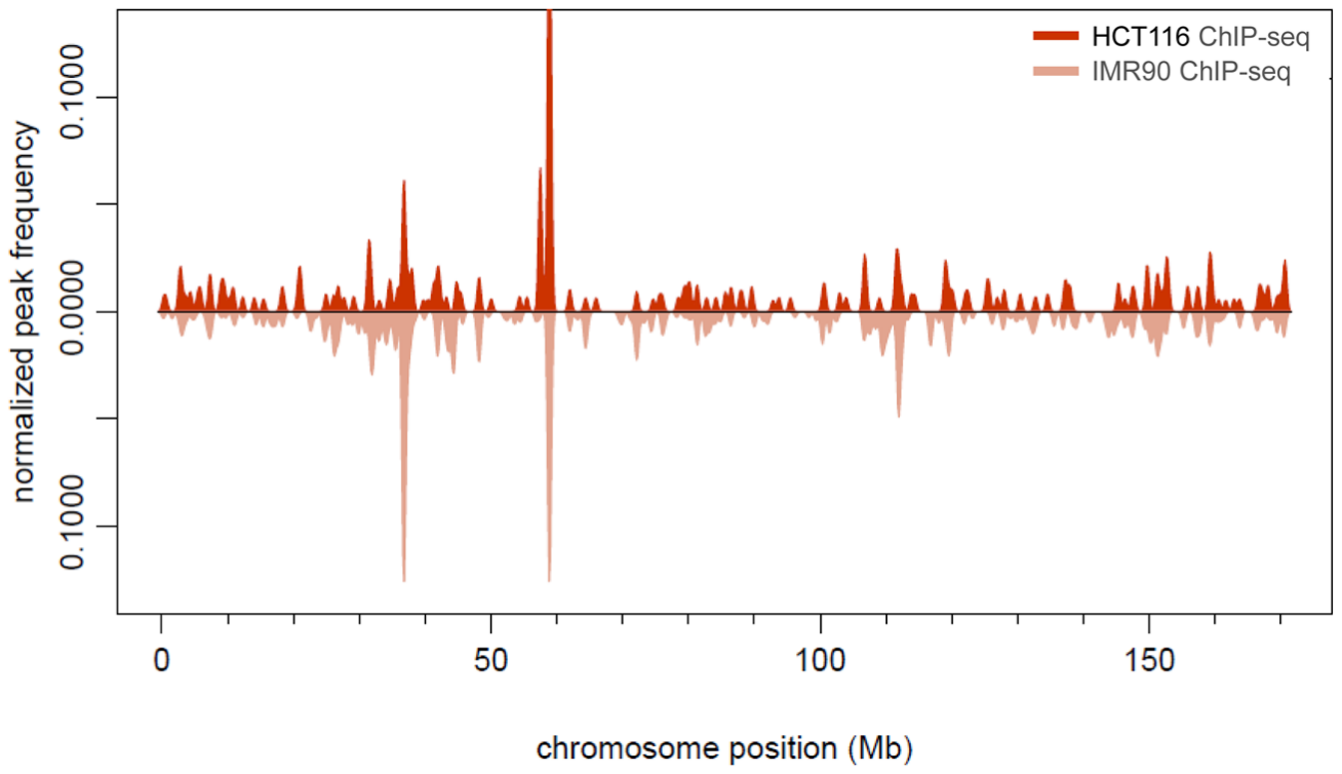
p53 Distribution of p53 ChIP-seq peaks on chromosome 6 in the cell lines HCT116 (this study) and IMR90 [16]. Plotted is peak frequency (per Mb) normalized by number of peaks in chromosome, smoothed by a Gaussian kernel density, peak-height weighted.

Overall, in HCT116 cells, the p53 genomic binding was less enriched at gene-rich-regions, depleted from CGIs, and more enriched at repeats, compared to IMR90 cells (Figure 5). These differences appear p53 specific, since they are not observed between the Inp-seq control datasets from the two cell lines. The global p53 binding profiles for chromosome 6 show regions of significant differences between HCT116 and IMR90 (Figure 6). Building up such maps under normal and stress conditions, and correlating them with matching chromatin state maps [23], could contribute to our understanding of the specificity of p53 genomic binding in the context of chromatin and the correlation with the functional elements in the human genome.

### p53 enrichment at repeats

**Figure 7:**
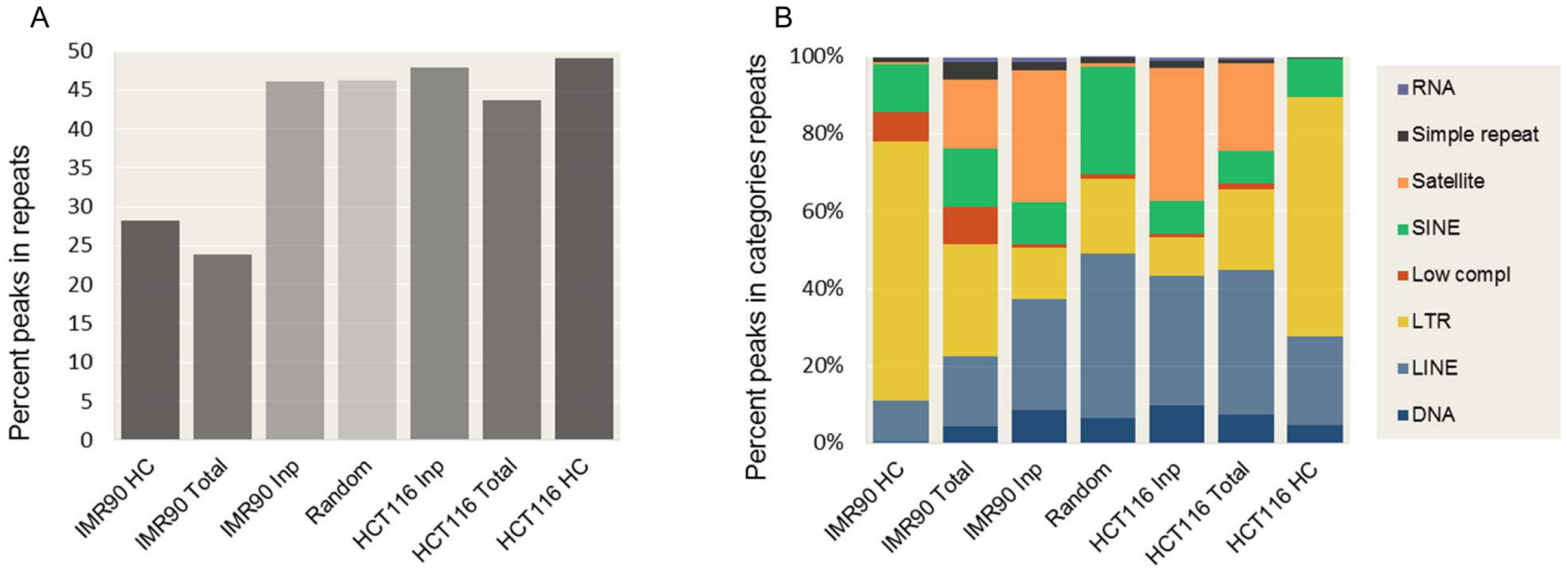
p53 enrichment in repeats. A) Fraction of p53 ChIP-seq and Inp-seq peaks in repeats. B) Repeat categories identified by RepeatMasker in the seven datasets analyzed: HCT116 HC (550 high-confidence p53 ChIP-seq peaks); HCT116 Total (3750 total p53 ChIP-seq peaks); HCT116 Inp (2168 Input-seq peaks); IMR90 HC (743 high-confidence p53 ChIP-seq peaks); IMR90 Total (6789 total p53 ChIP-seq peaks); IMR90 Inp (2550 Input-seq peaks) and Random (6789 random genomic locations, hg18). HCT116 data (this study); IMR90 data [16].

Repeats constitute a significant portion of the human genome [24],[25] and changes in p53 binding at repeats (Figure 5) could have a major impact on the entire network. Therefore, we looked in more details at the distribution and the types of repeats bound by p53. We correlated the p53 ChIP-seq peaks positions with the annotated human repeats (UCSC Human Genome Browser, as defined by Repbase [26]) for both the HCT116 (this study) and the IMR90 [16] datasets. Since the high-confidence p53 ChIP-seq peaks (550 HCT116, 743 IMR90) represent only small fractions of the total number of ChIP-seq peaks detected (3750 HCT116, 6789 IMR90), we analyzed both, to discriminate between features specific for the high confidence sets only, and those common for the entire datasets. The Inp-seq peaks (2168 HCT116, 2550 IMR90) were analyzed to examine the repeats representation in the chromatin from the two cell lines before the ChIP procedure. Since Inp-seq datasets do not represent entirely random genomic locations and are associated with significant biases [27]–[30], an independent control set was used, composed of random locations, see Materials and Methods. Similar fractions of the Inp-seq peaks in HCT116 and IMR90 resided in repeats, not much different from the set of the random genomic locations, unlike the high-confidence p53 ChIP-seq peaks (Figure 7A). For both cell lines, the total sets of ChIP-seq peaks (before any confidence tests) recapitulated the behavior of the high-confidence sets. Notably, in HCT116 cells, similar fractions of the ChIP-seq (total or high confidence) and Inp-seq peaks, resided in repeats, same as the random genomic locations (Figure 7A), which led us to investigate whether there were any p53 specific differences (enrichment or depletion at particular repeats types) between the HCT116 ChIP-seq and Inp-seq sets. All repeats types, as defined by Repbase [26] and annotated at the UCSC Human Genome Browser, identified in the analyzed datasets, are reported in Figure 7B, revealing common and cell line specific trends. The Inp-seq datasets from the two cell lines showed similar repeats profiles, albeit different from the random genomic locations set. In both Inp-seq sets, SINE, LINE and LTR repeats were underrepresented, while satellite repeats were overrepresented compared to the random control set. In both cell lines, the total sets of p53 ChIP-seq peaks showed less satellite and more LTR repeats compared to the Inp-seq datasets. That trend, common for the two cell lines, continued in the high-confidence p53 ChIP-seq sets, showing further overrepresentation of LTR elements (Figure 7B). Besides these common trends, there were clear differences between the two cell lines. In the HCT116 cells p53 was more enriched at LINE repeats; the fraction of HCT116 ChIP-seq peaks in LINE repeats was twice that in IMR90. In the IMR90 cells p53 was enriched at low complexity repeats (mainly GC-rich sequences).

p53 is known to bind to LTR [31], LINE [32] and ALU [33],[34] repeats. What we show here is that p53 binding at these repeats is selective and depends on the cell context. Significantly higher fraction of p53 binding sites resided in repeats in HCT116 compared to IMR90 cells (Figure 7A). Among all repeats bound by p53 (Figure 7B), LTRs were the most highly enriched in both cell lines, followed by LINEs in HCT116 and by ALUs in IMR90 cells. These are p53 specific findings, since the two Inp-seq sets (sampling the repeats in the chromatin from the two cell lines before the ChIP), showed similar profiles. The enriched p53 ChIP-seq peaks at repeats (particularly LINEs) in the HCT116 cells is intriguing, when placed in the context of recent reports indicating epigenetic impact on the p53 genomic binding, since the epigenetic dysregulation at repeats (and LINEs in particular) has emerged as a major cancer landmark.

We proposed previously that epigenetic changes, particularly local DNA hypermethylation at CGIs and global genomic hypomethylation, might be the main contributing factor for the distinct p53 genomic binding profiles we observed in normal and cancer cell lines [16]. Another possibility suggested was that in cancer cells, certain p53 binding sites may contain methylated CpG dinucleotides that undergo tumorogenesis-associated deamination to selectively lose p53 responsiveness [35]. Subsequently, a mouse study revealed that in absence of wild type p53, DNA demethylation triggers repeats instability, followed by a massive apoptotic response [36],[37]. Cancer-related changes in DNA methylation have been extensively studied, following the initial report for substantial hypomethylation in cancers compared to their normal counterparts [38]. Now it is known that cancer-associated epigenetic changes are widespread, affecting gene promoters, CpG islands and shores [39]. Hypomethylation at LINE repeats (normally methylated) is associated with bad prognosis in colon cancer [40]. It is tempting to speculate that the enriched p53 binding detected at LINE repeats in the colorectal cancer cell line HCT116, might be due to cancer-associated LINE hypomethylation. The higher overall p53 enrichment at repeats in HCT116 cells may be linked to the mismatch repair defects due to the MLH1 mutation present in this cell line, and the associated with it microsatellite instability [41]. Although these attractive possibilities address cancer-associated features of HCT116 in contrast to the normal IMR90, these cell lines differ as well by origin; HCT116 was established from colon epithelia, while IMR90 was derived from lung fibroblasts. Thus, tissue specific epigenetic marks may also contribute to the observed p53 binding differences. Using model systems allowing stepwise examination of the natural progression from normal to transformed state could help to define precisely the p53 genome-wide binding changes and the network changes due to the process of cancer progression.

### p53 binding motif analysis

**Figure 8:**
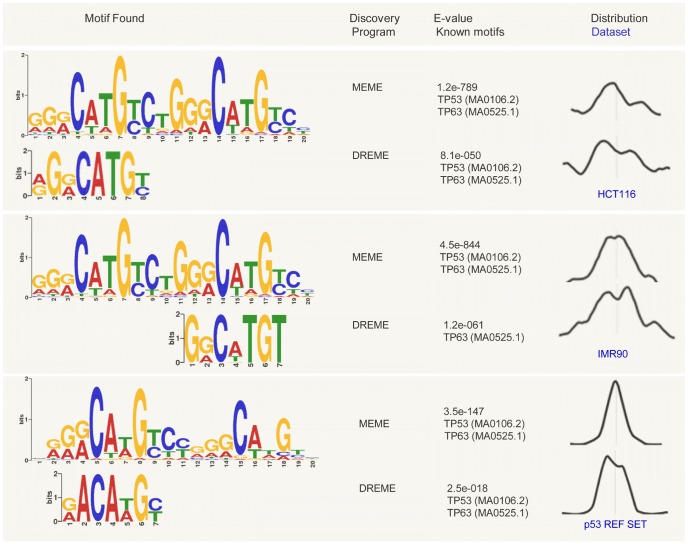
Sequencing logos depicting the statistically significantly enriched motifs identified in the high-confidence p53 ChIP-seq peaks in HCT116, in IMR90 [16], and in a set of 168 p53 reference sites (p53 REF SET), see Materials and Methods. Analysis was done with the MEME suite [42], using the programs MEME-ChIP [48] and DREME [46]. Shown is the distribution of the identified motifs in 100 nt windows centered at the peak maxima, reported by CentriMo [43].

Using the MEME suite [42] we looked for the most enriched sequence motifs in the high-confidence set of 550 p53 ChIP-seq peaks in HCT116 cells, and obtained nearly canonical p53 consensus sequence (MEME motif, 1.2e-789, Figure 8). All centrally enriched, statistically significant motifs identified in the HCT116 and IMR90 datasets were very similar (Figure 8). As a control dataset for this analysis we used 168 reference p53 binding sites, see Materials and Methods. Although containing a lower number of sequences than the ChIP-seq datasets, this control set has the advantage of being composed of individually defined, functional p53 REs. Keeping the analysis settings constant, we obtained similar motifs for the control dataset as for the ChIP-seq sets (Figure 8). The major difference observed was in the distribution of the motifs around the peak maxima, as reported by CentriMo [43]. The enriched p53 motifs in the HCT116 ChIP-seq dataset showed wider distribution, more distant from the peak maxima, followed by the p53 motifs in the IMR90 ChIP-seq dataset, and the control dataset, which displayed most tightly centered motifs (Figure 8). The three datasets showed similar fractions of sequences not recognized by MEME to contain p53 related motifs (although the control reference set was composed of proved functional p53 REs), fact pointing at algorithm limitations in motif calling.

### Distinct pathways associated with genes harboring p53 binding sites in and out of CpG islands

Given the distinct p53 genome-wide binding patterns in HCT116 and IMR90, we compared the signaling pathways enriched in these two cell lines, based on the genes harboring ChIP-seq peaks. For functional annotation of all linked gene ontology (GO) terms associated with the high-confidence ChIP-seq peaks in HCT116, DAVID Functional Clustering was used [44]. A total of 104 gene clusters were identified, and the most highly enriched 42 clusters are listed in Table S5.

**Figure 9:**
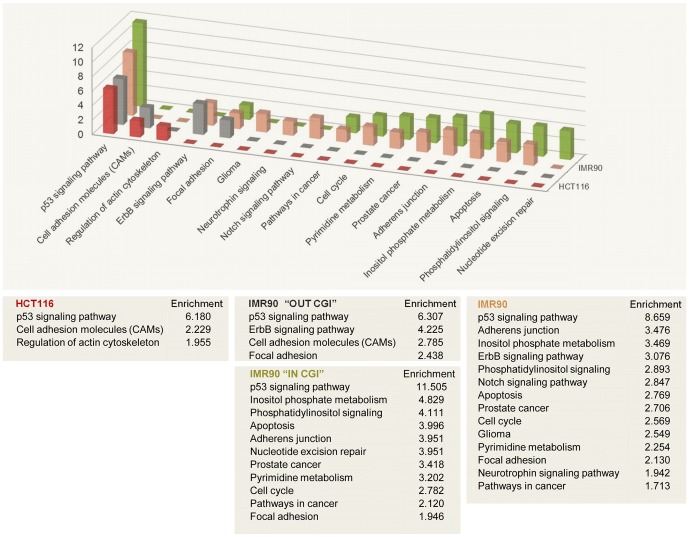
The most highly enriched pathways in HCT116 and IMR90 cells are distinct, demonstrated by DAVID GO analysis [44] of the genes associated with p53 ChIP-seq peaks in HCT116 (this study) and IMR90 [16]. Genes associated with IMR90 peaks in and out of CpG islands contribute to distinct pathways. Fold enrichment for each pathway is reported and plotted on the y-axis of the graph. Analyzed high-confidence p53 ChIP-seq sets: HCT116 (550 peaks), IMR90 (743 peaks), IMR90 IN CGI (331 peaks), IMR90 OUT CGI (412 peaks).

For comparing the most enriched signaling pathways, we used the genes associated with high-confidence ChIP-seq peaks in HCT116 (this study) and in IMR90 [16], DAVID Annotation Chart Analysis and the Kyoto Encyclopedia of Genes and Genomes (KEGG), see Materials and Methods. Because the overlap between the HCT116 and IMR90 p53 ChIP-seq peaks was due mainly to the IMR90 peaks out of CGIs (Figure 2A), we examined separately the two IMR90 subsets: genes associated with peaks in CGIs, and genes associated with peaks out of CGIs. All statistically significant enriched pathways in the analyzed four datasets are shown in Figure 9, as reported by DAVID, without manual curating. The only common pathway for all four is the p53 signaling pathway, detected as the most highly enriched pathway in each (Figure 9). Both quantitatively (by the number of pathways detected) and qualitatively (by the types of functions affected), the HCT116 dataset was similar to the IMR “out of CGIs” dataset, and very different from the IMR90 “in CGIs” dataset. The majority of the signaling pathways detected in the IMR90 datasets are missing from the HCT116 dataset because they are due to the ChIP-seq peaks in CGIs. In fact, the two IMR90 subsets (p53 ChIP-seq peaks “in CGIs” and “out of CGIs”) show distinct enriched signaling pathways with almost no overlap between them (besides the p53 pathway itself), indicating that the distinct p53 genomic binding profiles translate into distinct functional types of genes bound by p53, contributing to distinct putative signaling pathways. Our data support the model for p53 binding to the human genome in a highly selective manner, affecting distinct sets of genes, contributing to distinct functional pathways.

## Conclusions

While the p53 ability to evoke stress-specific and cell-type-specific responses is well recognized [45], it remains to be defined how that specificity is established. Whether p53 binds to the genome in a selective manner is still an open question. Previously, we reported distinct p53 genomic binding patterns based on analysis of data obtained by us in the normal human cell line IMR90 and by others in the cancer cell lines HCT116 and U2OS [16]. Many variables were associated with these datasets (cell lines, treatments, experimental approaches), all potentially contributing to the observed differences. Here, we mapped the p53 binding sites in HCT116 cell line under conditions matching our previous IMR90 study. This study compared *de novo* high-resolution p53 genome-wide binding maps in two cell lines under otherwise identical conditions (treatment, approach, analysis pipeline). Not only did we prove that under the same conditions IMR90 and HCT116 have different p53 binding profiles, but we showed that starting with the same cell line and treatment (HCT116, 6 hrs 5 FU), despite the different experimental approaches (ChIP-seq vs ChIP-PET), the end result was remarkable similarity in the p53 binding profiles. The conclusion from this study is that p53 genomic binding is selective and cell context dependent. Based on this study, our previous data [16] and reports by others [36], our interpretation of the observed p53 binding differences is that they reflect the cancer–associated epigenetic changes accumulated in HCT116 cells (absent from IMR90), superimposed on tissue specific differences (HCT116 has epithelial, while IMR90 has mesenchymal origin). Defining the origin of p53 binding selectivity by dissecting cancer-associated from tissue specific differences, would help to understand the fundamental mechanisms regulating p53 interactions with the human genome in the context of chromatin and changing epigenome.

## Materials and Methods

### Cell cultures and treatments

The human colorectal cancer cell line HCT116 was obtained from ATCC (CCL-247). Cells were maintained in McCoy's 5A Medium (Invitrogen) supplemented with 10% fetal bovine serum. Cultures were grown at 37°C in a humidified atmosphere containing 5% CO_2_. Cells were treated with 5-FU (SIGMA) for 6 hrs at a final concentration of 375 µM. Whole cell extracts from HCT116 cells were subjected to Western analysis using the p53-specific antibody DO1 (sc-126, Santa Cruz). Quantitative PCR was performed on Rotor-Gene 3000 (Roche) using SYBR Green PCR Master Mix (Applied Biosystems), target-specific primers (Integrated DNA Technologies) and 2 µl ChIP DNA as a template. Enrichment in the ChIP samples at specific target sites was calculated as a fraction of the total Input (%).

### Chromatin immunoprecipitation and ChIP-seq library preparation

The ChIP experiments were done with the p53-specific antibody DO1 (sc125X, Santa Cruz) using normal mouse IgGs (sc2025, Santa Cruz) for control. Experimental protocols for the ChIP, ChIP-seq and Input-seq libraries preparations were as described [16]. Briefly, the cells were treated for 6 hrs with 5-FU, fixed with 1% formaldehyde, harvested, lysed and the chromatin was sonicated. Input (chromatin sample) was taken before the immunoprecipitation, The antibody-chromatin complexes were purified, and the cross-links reversed at 65°C for 4 hr. DNA samples (ChIP and Input) were incubated with RNaseA, Proteinase K, followed by phenol/chloroform extraction and QIAGEN PCR clean up. ChIP and Input DNA libraries for single-end sequencing were prepared as described [16], after polishing and Illumina adapter ligation, ChIP and Input DNAs were PCR amplified, gel purified and sequenced on Illumina (GA IIx).

### Data Analysis

Illumina 36 nt sequencing reads were mapped to the reference human genome (hg18) using in-house developed software and the genomic locations enriched for p53 were identified by adapting published methods [18] as described [16]. Distinct sequence reads mapped uniquely (one mismatch allowed per 36 nt read) were used for building up coverage maps at each nucleotide in the human genome, and locations with ChIP-seq reads above the assigned threshold were identified. These were then filtered by applying statistical confidence tests, requiring enrichment over the Input-seq reads to correct for systematic biases present in the data. High-confidence peak locations were entered into an SQL database for positional correlation with various genomic features (RefSeq genes, CGIs, repeats etc.) as described [16], see below.

### p53MH sites

A local copy of the p53MH program [21] was used to identify putative p53 binding sites (score 75% and above) in 2 kb intervals centered on the peak maxima. All predicted p53MH sites found within +/−100 of the peak maxima are reported in Table S2.

### TSS and Refseq genes

Proximity to TSSs was analyzed by examining 10 kb regions centered at ChIP-seq peak maxima (Figure 3). All TSSs identified (UCSC Genome Browser) were plotted as a function of distance to peak maxima. All RefSeq genes, where the peak maxima occurred within 20 kb of either end of the gene or inside (exon + introns), are listed in Table S2. Peaks are reported close to single gene if found in proximity to a single gene/transcript, and close to multiple genes – if in proximity to more than one gene/transcript (Figure 5). Proximity is defined as a peak maximum location within 20 kb upstream of TSS, inside a gene, or up to 5 kb downstream of TES.

### CpG islands

ChIP-seq peaks were reported to be associated with CGIs (UCSC Human Genome Browser) if peak maxima were located within CGI boundaries extended by 350 nt (half the average size of chromatin fragments used in the study).

### Repeats

Repeats were identified using RepeatMasker at http://repeatmasker.org and Repbase [26], Genetic Information Research Institute (http://www.girinst.org/). ChIP-seq and Inp-seq peaks were classified to be in repeats if peak maxima were inside the repeats boundaries (as annotated, UCSC Human Genome Browser). Seven datasets were analyzed: high-confidence p53 ChIP-seq peaks (550 in HCT116; 743 in IMR90); total p53 ChIP-seq peaks (3750 in HCT116; 6789 in IMR90); total Inp-seq peaks (2168 in HCT116; 2550 in IMR90). The seventh set, used as an independent control, was composed of 6789 random genomic locations (random points were chosen in hg18 by Monte Carlo, then extended in both directions to obtain sequences matching the average size of the ChIP fragments used in the study; the number 6789 was chosen to match the experimental dataset with the highest number of sequences).

Examples of types of repeats reported in Figure 7, as identified by RepeatMasker: RNA (rRNA, snRNA, tRNA); Simple repeats (TG)n, (TCC)n, (CACTC)n, (GAGTG)n, (TATATG)n; Satellite Repeats (Satellite/acro ACRO1, Satellite/centr ALR/Alpha, Satellite/telo REP522); SINE (SINE/Alu, SINE/Deu, SINE/MIR); Low Complexity (C-rich, GC-rich, GA-rich, CT-rich); LTR (LTR/ERV1, LTR/ERVK, LTR/ERVL, LTR/Gypsy); LINE (LINE/CR1, LINE/L1, LINE/L2); DNA (DNA/MuDR, DNA/PiggyBac, DNA/TcMar-Mariner, DNA/hAT-Charlie).

### ChIP-PET clusters

The ChIP-PET data [8] were downloaded from the UCSC Human Genome Browser, and the clusters were reconstructed. For analyses were used all clusters ChIP-PET1+ to ChIP-PET7+, as described [8], where the cluster rank corresponds to the number of overlapping PET fragments (e.g. PET3+ cluster is composed of at least 3 overlapping PET fragments). For comparison with the ChIP-seq peaks, the ChIP-PET clusters were reported by rank. A ChIP-seq peak was reported overlapping a ChIP-PET cluster if the peak maximum was within 350 nt (half the average chromatin fragments size) of the cluster boundaries, criteria previously validated [16]. For comparing peak heights at the 189 locations common for the HCT116 and IMR90 cell lines (Figure 2D and 2E), normalized peak values were used (shown as percent from the highest peak in the set); peaks were ordered first by peak height in HCT116, then by peak height in IMR90 (Figure 2C), or first by peak height in IMR90, then by peak height in HCT116 (Figure 2D).

### Reference p53 REs

A set of 168 individually analyzed reference p53 REs [16] was used as a positive control for analysis of significantly enriched motifs (Figure 8). The midpoint of each RE was extended with 50 nt in both directions to obtain a set of 168 sequences matching by size the 100 nt ChIP-seq sequences used for the p53 motif analysis (see Motif Discovery).

### Motif Discovery

*De novo* motif analysis was done with MEME 4.6.1. [42], and DREME [46], using the high-confidence set of 550 p53 ChIP-seq peaks identified in HCT116. For comparison, the previously reported high-confidence set of 743 p53 ChIP-seq peaks in IMR90 cells [16] was used. Motif searching was done on 100 nt sequences centered at the peak maxima and the settings were kept constant for all datasets (including the positive control p53 REF set, see above). The statistically significantly enriched motifs (identified by MEME or DREME) were searched for known transcription factors using TOMTOM and the databases JASPAR and UniPROBE [47].

### Functional Annotation

Identification of the most enriched pathways based on the genes associated with p53 ChIP-seq peaks in HCT116 was done with DAVID 6.7. [44] A ChIP-seq peak was considered gene associated if the peak maximum was inside a gene (exon or intron), or within 20 kb of gene ends. Most enriched pathways were determined using DAVID Annotation Chart Analysis and Kyoto Encyclopedia of Genes and Genomes (KEGG) database. For comparison with the pathways enriched in IMR90 cells, the previously reported list of genes associated with the 743 high-confidence p53 ChIP-seq peaks in IMR90 cells [16] was used. Two additional subsets were analyzed, one of genes associated with IMR90 peaks in CpG islands (IN CGIs, 331 peaks) and another of genes associated with IMR90 peaks out of CpG islands (OUT CGIs, 412 peaks).

## Supporting Information

Figure S1 **p53 activation in HCT116 cells after 6hrs treatment with 5FU.** **S1A.** Whole cell extracts from HCT116 cells, treated for 6 hrs with 5-FU, were subjected to Western analysis using the p53-specific antibody DO1 (sc-126). Actin was used as a loading control. **S1B.** p53 enrichment was confirmed by qPCR at the target genes *CDKN1A* and *MDM2*. *CDKN1A* site: -2,232 bp to TSS (chr6:36,752,204-36,752,224); *MDM2* site: chr12: 67,488,970-67,488,990; negative control for p53 binding: chr22:47,056,575-47,056,854. Coordinates are given in hg18. Average enrichment is calculated as percentage of the total Input; shown are results from duplicated measurements. FU (6 hrs treatment with 5-FU); NS (no stimulation); DO1 (ChIP with p53-specific DO1 antibody); IgG (mock ChIP with non-specific IgG).
(PDF)

Figure S2 **Defining high-confidence p53 ChIP-seq peaks in HCT116.**
(PDF)

Table S1 **Sequencing runs statistics.**
(XLSX)

Table S2 **List of 550 high-confidence p53 ChIP-seq peaks identified in HCT116 cells after 6rhs treatment with 5 FU.**
(XLSX)

Table S3 **List of 189 high-confidence HCT116 p53 ChIP-seq peaks overlapping high-confidence IMR90 p53 ChIP-seq peaks.**
(XLSX)

Table S4 **Distribution of the ChIP-seq peaks with respect to genes in HCT116 cells.**
(XLSX)

Table S5 **Functional annotation clustering of the genes associated with the 550 high-confidence p53 ChIP-seq peaks in HCT116 cells.**
(XLSX)
