# Supplementary material for: Cell Context Dependent p53 Genome-Wide Binding Patterns and Enrichment at Repeats
Source: PLoS One. 2014 Nov 21;9(11):e113492. doi: 10.1371/journal.pone.0113492 (PMC4240582; doi:10.1371/journal.pone.0113492)
Supplement: Figure S1 — p53 activation in HCT116 cells after 6hrs treatment with 5FU. S1A. Whole cell extracts from HCT116 cells, treated for 6 hrs with 5-FU, were subjected to Western analysis using the p53-specific antibody DO1 (sc-126). Actin was used as a loading control. S1B. p53 enrichment was confirmed by qPCR at the target genes CDKN1A and MDM2. CDKN1A site: -2,232 bp to TSS (chr6:36,752,204-36,752,224); MDM2 site: chr12: 67,488,970-67,488,990; negative control for p53 binding: chr22:47,056,575-47,056,854. Coordinates are given in hg18. Average enrichment is calculated as percentage of the total Input; shown are results from duplicated measurements. FU (6 hrs treatment with 5-FU); NS (no stimulation); DO1 (ChIP with p53-specific DO1 antibody); IgG (mock ChIP with non-specific IgG). (PDF) [file pone.0113492.s001.pdf]

S1A S1B

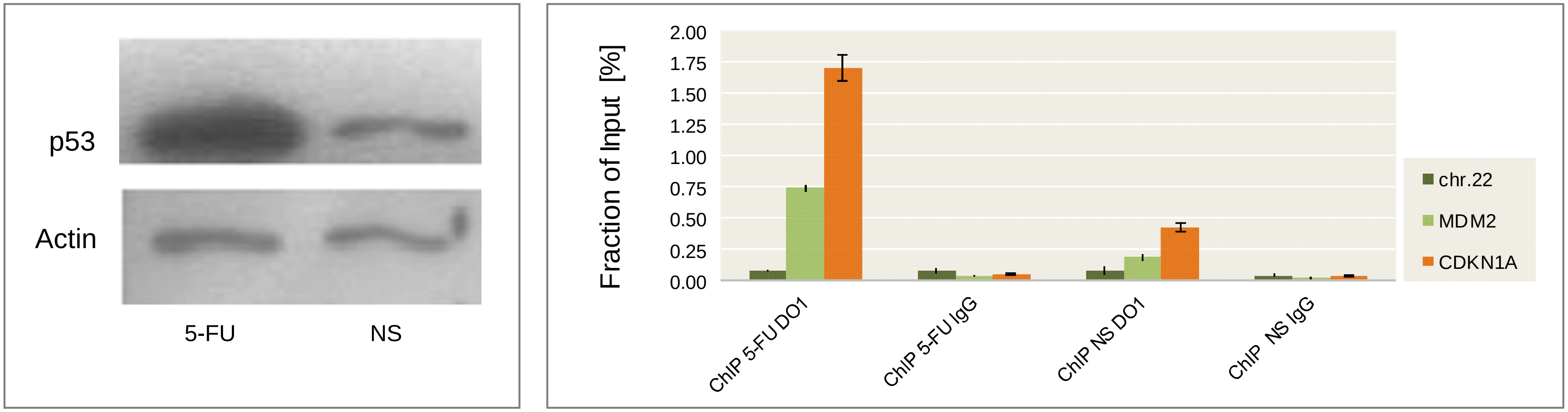

Figure S1. p53 activation in HCT116 cells after 6hrs treatment with 5FU.

S1A. Whole cell extracts from HCT116 cells, treated for 6hrs with 5-FU, were subjected to Western analysis using the p53-specific antibody DO1 (sc-126). Actin was used as a loading control.

S1B. p53 enrichment was confirmed by qPCR at the target genes *CDKN1A* and *MDM2*. *CDKN1A* site: -2,232 bp to TSS (chr6:36,752,204-36,752,224); *MDM2* site: chr12: 67,488,970-67,488,990; negative control for p53 binding: chr22:47,056,575-47,056,854. Coordinates are given in hg18.

Average enrichment is calculated as percentage of the total Input; shown are results from duplicated measurements. FU (6hrs treatment with 5-FU); NS (no stimulation); DO1 (ChIP with p53-specific DO1 antibody); IgG (mock ChIP with non-specific IgG).
