# Supplementary figures and images for: Cell Context Dependent p53 Genome-Wide Binding Patterns and Enrichment at Repeats
Source: PLoS One. 2014 Nov 21;9(11):e113492. doi: 10.1371/journal.pone.0113492 (PMC4240582; doi:10.1371/journal.pone.0113492)

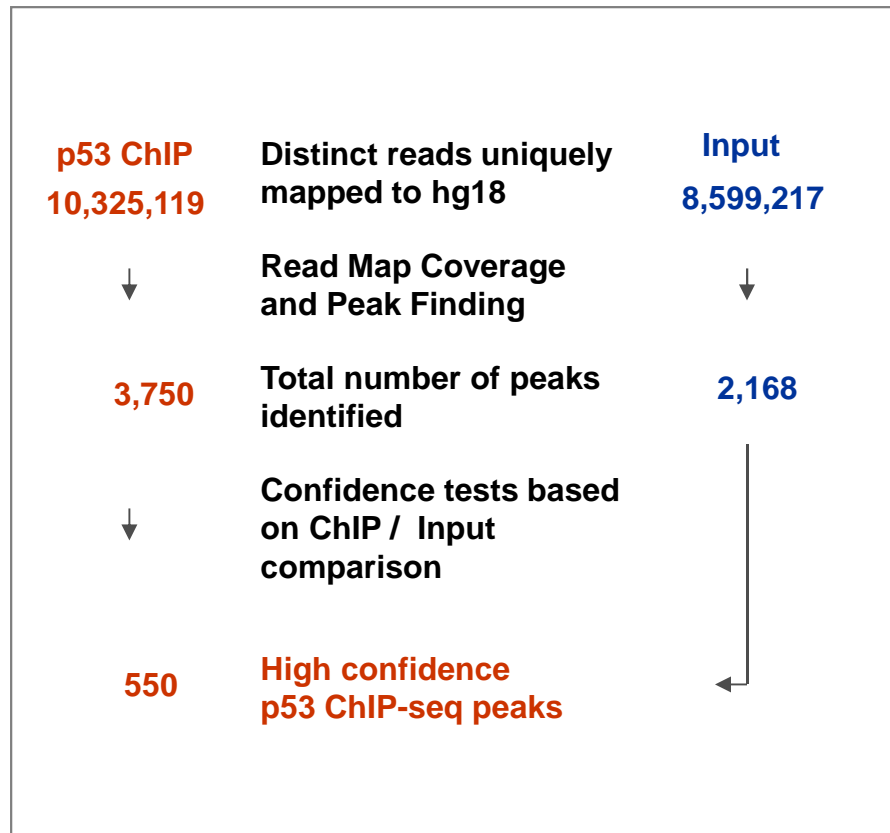

**Figure S2. Defining high-confidence p53 ChIP-seq peaks in HCT116.**

Supplement: Figure S2 — Defining high-confidence p53 ChIP-seq peaks in HCT116. (PDF) [file pone.0113492.s002.pdf]
